# LncRNA *GATA3-AS1* Promotes Bladder Uroepithelial Cancer Progression by Stabilizing DDX5

**DOI:** 10.7150/jca.104034

**Published:** 2025-01-01

**Authors:** Yatao Duan, Zonghang Liu, Yongliang Ma, Pan Qi, Yanli Guo, Aili Zhang

**Affiliations:** 1 Department of Urology, The Fourth Hospital of Hebei Medical University, Shijiazhuang, 050000, Hebei, China.; 2 Hebei Cancer Institute, The Fourth Hospital of Hebei Medical University, Shijiazhuang, 050000, Hebei, China.

**Keywords:** bladder urothelial carcinoma, *GATA3-AS1*, DDX5, Wnt/β-catenin

## Abstract

**Objective:** Exploration of molecular markers is an ongoing focus in the field of bladder cancer research. Based on data from public databases, *GATA3-AS1* was identified as upregulated in bladder urothelial carcinoma (BLCA); however, its exact function and regulatory mechanism in this context remain unclear.

**Methods:** To investigate the clinical implications of *GATA3-AS1*, we examined its levels in 90 BLCA and adjoining normal tissue samples. Functional assays were conducted to assess the effects of *GATA3-AS1* on BLCA cell proliferation, migration, and invasion. Animal assays were employed to determine the effects of *GATA3-AS1* on BLCA tumorigenicity *in vivo*. Immunoblotting, RNA pull-down, RNA immunoprecipitation, TOP/FOP luciferase reporter gene, and coimmunoprecipitation assays were used to explore the molecular mechanism underlying the effects of *GATA3-AS1* on BLCA progression.

**Results:** *GATA3-AS1* expression was significantly up-regulated in BLCA tissues and correlated with pathological stage, grade, and poor patient outcome. Altered *GATA3-AS1* levels promoted BLCA proliferation, migration, and invasion. Mechanistic studies suggested that *GATA3-AS1* interacts with DDX5 protein, enhances its stability, and ultimately leads to BLCA progression through Wnt/β-catenin signaling pathway activation.

**Conclusion:** *GATA3-AS1* overexpression increases the aggressiveness of BLCA by activating the Wnt/β-catenin pathway through binding to DDX5. *GATA3-AS1* has potential as a new molecular predictor of poor prognosis in patients with BLCA.

## Introduction

Bladder cancer is the tenth most common cancer worldwide and the second most frequently occurring malignant tumor of the urinary system, representing a severe threat to human health and placing a heavy burden on healthcare^1^, ^2^. Bladder urothelial carcinoma (BLCA) is the most predominant histological type of bladder cancer, comprising approximately 90% of all bladder cancers^3^, among which 70%-75% are non-muscle invasive bladder cancers (NMIBCs), while 20%-25% of patients with BLCA have invasion of the muscle layer^4^. Despite transurethral resection of bladder tumors and risk-stratified intravesical adjuvant bladder therapy for NMIBC, disease will recur in 40% of patients and 15%-20% will develop muscle-invasive bladder cancer^5^-^7^. Even with aggressive treatment, almost 50% of muscle-invasive bladder cancer cases will develop distant metastases within 3 years, resulting in a low 5-year survival rate^8^. Bladder cancer is highly heterogeneous, both histologically and clinically, and bladder cancers with diverse molecular characteristics can develop into different histological subtypes that exhibit distinct biological features, ultimately leading to varying treatment outcomes^9^. Molecular subtypes have been the focus of bladder cancer research in the past decade. Although several subtype systems are currently promising, in terms of their associations with clinical outcomes and treatment responses, their clinical value has yet to be established^10^. Therefore, continuous exploration to identify biomarkers for BLCA remains warranted.

Long non-coding RNAs (lncRNAs) cannot be translated into proteins, but can regulate gene expression and protein function by interacting with DNA, RNA, and proteins, thus acting as oncogenes or tumor suppressor genes^11^, ^12^. There is increasing evidence that lncRNAs are involved in the regulation of cell proliferation, migration, invasion, apoptosis, epithelial-mesenchymal transition (EMT), and energy metabolism during tumorigenesis and cancer progression^13^-^16^. Aberrant expression of lncRNAs also plays a regulatory role in BLCA, and is closely correlated with various clinical features of BLCA, including tumor size, TNM stage, and patient prognosis^17^, ^18^.

GATA-binding protein 3 antisense RNA 1 (*GATA3-AS1*) is an antisense lncRNA for GATA-binding protein 3 (GATA3) that can act as an oncogene in various cancers. For example, *GATA3-AS1* facilitates triple-negative breast carcinoma tumor progression and immune escape by promoting GATA3 protein ubiquitination and up-regulating COPS5, to stimulate deubiquitination of PD-L1^19^. Further, *GATA3-AS1* promotes endometrial carcinoma development through competitive binding to *miR-361*, up-regulation of ARRB2, and subsequent stimulation of the Src/Akt pathway^20^. In addition, *GATA3-AS1* affects pancreatic carcinogenesis by sponging *miR-30b-5p* to regulate Tex10, which may be relevant to the Wnt/β-catenin signaling pathway^21^. Using data from public databases, we identified abnormal elevation of *GATA3-AS1* expression in BLCA and hypothesized that *GATA3-AS1* contributes to BLCA genesis and progression. In this research, we analyzed *GATA3-AS1* expression in BLCA and its relationship with clinical features of the disease, as well as revealing the downstream molecular mechanisms by which *GATA3-AS1* regulates BLCA cell invasion and metastasis.

## Materials and Methods

### Patients and specimens

Tumor and adjacent matching normal tissue samples from 90 patients with BLCA who underwent surgical treatment were provided by the Biological Sample Bank of Hebei Provincial Institute of Cancer Research. Informed consent was obtained from all patients before sample collection and this study was approved by the Ethics Committee of the Fourth Hospital of Hebei Medical University. No patients underwent any type of antitumor therapy before surgery.

### Cell culture

Cells were obtained from ATCC. Human BLCA cell lines (T24, 5637, SW780) were cultured in RPMI 1640 medium (Invitrogen), and immortalized human bladder epithelial cells (SV-HUC-1) were cultured in Ham's F12K medium (Gibco); both types of media were supplemented with 10% fetal bovine serum (Invitrogen). Cells were cultured in an incubator at 37°C with 5% CO_2_.

### RNA extraction and quantitative real-time PCR (qRT-PCR)

Cells or tissues were lysed using Triquick reagent (Solarbio, Beijing, China), and then total RNA was extracted using chloroform. cDNA synthesis was performed using All-In-One 5× RT Master Mix (Applied Biological Materials Inc., Canada). RT-qPCR was performed using qPCR premix (Yeasen, China). Target gene expression was quantified using the 2^-ΔΔCT^ method, relative to levels of *GAPDH*. Primer sequences are listed in Supplementary Table 1.

### Cell transient transfection and construction of stable cell lines

Plasmids containing *GATA3-AS1*, *DDX5*, and negative control sequences in the pcDNA3.1+ vector were synthesized by GenScript (Nanjing, China). GenePharma (Suzhou, China) provided an antisense oligonucleotide (ASO) targeting *GATA3-AS1*, siRNA targeting *DDX5*, and the corresponding negative control; sequences of ASO RNA and siRNA are presented in Supplementary Table 2. Transient transfection was conducted using Lipofectamine 2000 (Invitrogen, USA), and RNA and protein samples were collected after 24 and 48 h, respectively. To obtain stable overexpressing cell lines, lentiviral infection and puromycin (1 μg/ml) screening were performed in T24 cells, using standard protocols.

### MTS assay

Cells are inoculated into a 96-well plate (1000 cells/well) and MTS reagent (Promega, USA) is added to a group of wells every 24 h and the absorbance at 490 nm is measured for that group of wells.

### Colony formation assay

Cells were inoculated in six-well culture plates (1500 cells per well), cultured for 7 days, fixed with 4% paraformaldehyde, stained with 0.1% crystal violet, and finally counted as colonies.

### Transwell invasion assay

Cells and serum-free medium were added to Matrigel gel-lined Transwell chambers (Nest Biotech, China), and serum-containing medium added to the lower chambers. After incubation for 24 h, cells were fixed, stained, and counted under a microscope.

### Wound healing assay

Cells grown to confluence in six-well plates were scratched using a 200 µl pipette tip. Images were then captured under a microscope immediately and again 24 h later, and the rate of cell migration into the scratch calculated.

### Fluorescence in-situ hybridization (FISH)

A digoxigenin-labelled oligonucleotide probe for GATA3-AS1 was obtained from BOSTER Biotech; the sequences were: 5'-CGTCAGAAACGCTGCGGATGCCAGGTCTTGAAAATGCTGA-3' and 5'-GAGGCTAAGAATTATTTCAAAGACAAAAAGAAAGACTGG-3'. Nuclei were stained with DAPI and observed under a fluorescence microscope, following the steps in the product manual.

### Western blotting

Protein samples were prepared using RIPA rapid lysis buffer (Solarbio, China), separated by 10% SDS-PAGE and transferred to PVDF membranes. The PVDF membrane was blocked and then incubated with primary and secondary antibodies. ECL chemiluminescent solution was then added to develop protein bands. Primary antibodies were as follows: anti-DDX5 (R24094, ZenBio), anti-β-catenin (R22820), anti-E-cadherin (R22490), anti-N-cadherin (R380671), anti-Vimentin (R22775), anti-β-actin (R380624), anti-myc (ab32072, Abcam), anti-cyclinD1 (ab134175), and anti-Axin2 (ab32197).

### TOP/FOP luciferase reporter gene assay

Cells were co-transfected with TOP/FOP Flash reporter plasmid (Shanghai Beyotime, China) and overexpression plasmids or siRNA. Luciferase activity was assayed after 48 h.

### RNA pull-down assay

Proteins were pulled down with RNA probes containing the *GATA3-AS1* positive or negative sequence, using a Pierce Magnetic RNA-Protein Pull-Down Kit (Thermo), following the manufacturer's instructions. RNA-binding proteins (RBPs) were analyzed by silver staining and mass spectrometry (MS), and the target proteins confirmed by western blot.

### RNA-protein immunoprecipitation (RIP) and co-immunoprecipitation (Co-IP) assays

After treatment of cells with immunoprecipitation lysis buffer containing PMSF, supernatants were collected, and RNA or protein bound to DDX5 protein enriched using A/G beads conjugated to anti-DDX5 antibody. For Co-IP, anti-DDX5 antibody was incubated with IP samples at 4°C overnight. Immunoprecipitation complexes were then enriched using magnetic beads and analyzed by western blot.

### Animal experiments

Animal experiments were approved by the Animal Committee of the Fourth Hospital of Hebei Medical University. Stably transfected T24 cells overexpressing *GATA3-AS1* and negative control cells were injected subcutaneously into five 6-week-old male BALB/c nude mice (5×10^6^/each). Tumor volumes were measured and recorded every 7 days until mice were euthanized after 4 weeks.

### Statistical analysis

Each *in vitro* experiment was repeated independently three times, and the results are expressed as the mean ± standard deviation. The student's t-test was used to compare means between groups, and the chi-square test was used to assess associations between levels of *GATA3-AS1* and clinicopathological factors. Survival curves were plotted using the Kaplan-Meier method, and analyzed using the log-rank test. P < 0.05 was considered significant.

## Results

### *GATA3-AS1* is upregulated and associated with clinical characteristics in BLCA

**Figure 1:**
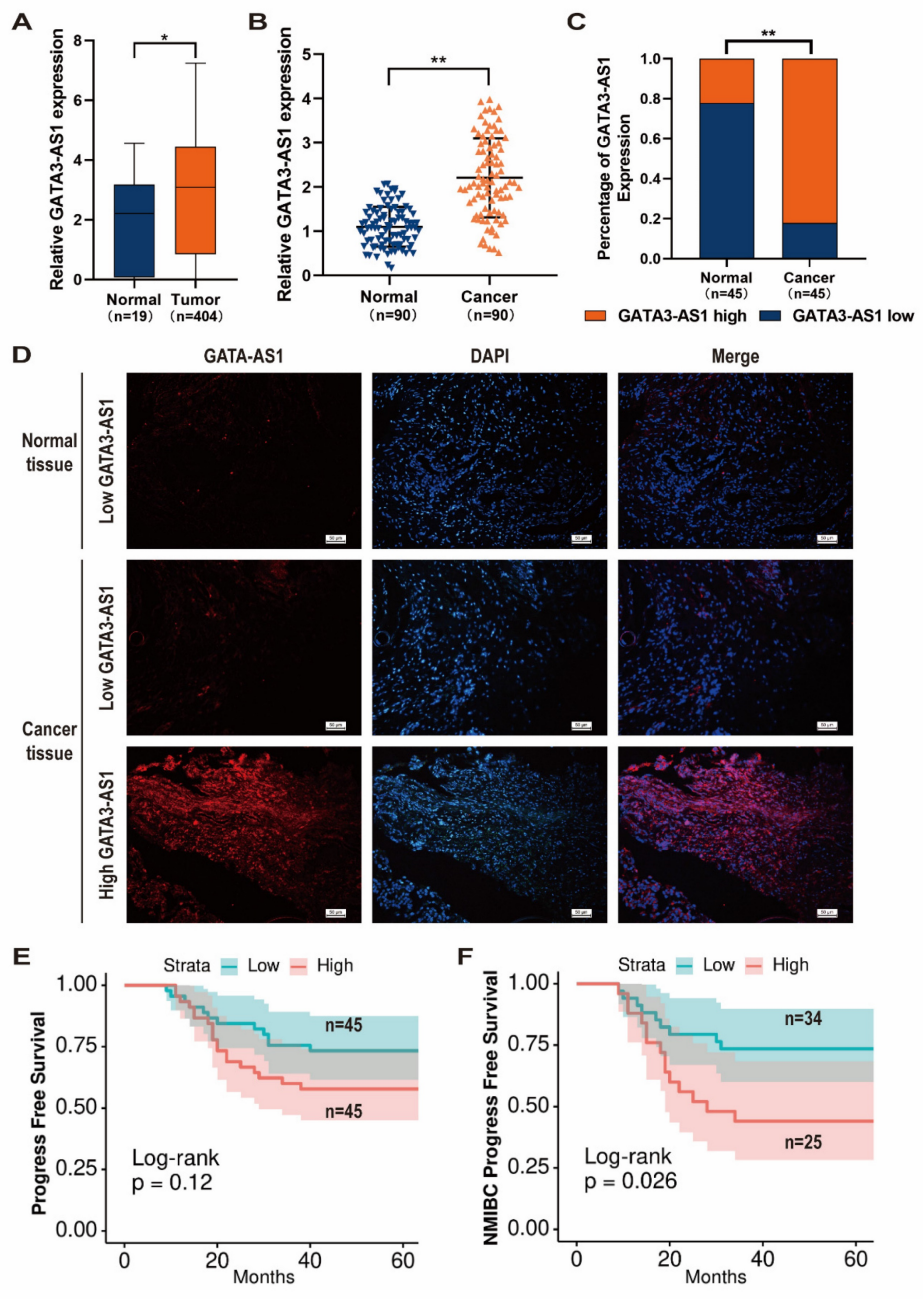
***GATA3-AS1* expression upregulated in BLCA and predicts poor prognosis.** (**A**) Expression levels of GATA3-AS1 in 404 bladder tumor samples and 19 normal controls from TCGA cohort. (**B**) The expression levels of *GATA3-AS1* in 90 pairs of BLCA tissues and adjacent normal tissues were detected by RT-qPCR method. (**C-D**) Representative images (**D**) and statistical analysis (**C**) of RNA FISH staining of *GATA3-AS1* in 45 paired BLCA tissues and adjacent normal tissues. Nuclei are stained with DAPI. (**E**) Kaplan-Meier curves of *GATA3-AS1* in 90 BLCA patients for progress free survival (PFS). (**F**) Kaplan-Meier curves of *GATA3-AS1* in non-muscle invasive bladder cancer (NMIBC) patients for PFS. Data are shown as mean ± SD from three independent experiments. ^*^P < 0.05, ^**^P < 0.01.

Using data from TCGA database, we identified *GATA3-AS1* as having abnormally elevated expression in BLCA tissues compared with normal bladder mucosa (Figure 1A). To confirm this finding, we performed RT-qPCR analysis of samples from 90 patients with BLCA. In addition, we randomly selected 45 samples for FISH analysis. Both assays confirmed that *GATA3-AS1* expression was up-regulated in BLCA (Figure 1B-D).

Next, patients were categorized into high (n = 45) and low (n = 45)* GATA3-AS1* expression groups, based on median values from RT-qPCR data, and associations between *GATA3-AS1* levels and BLCA clinical features analyzed. Patients with high *GATA3-AS1* expression were more likely to develop myxoid infiltration and have high pathological grade tumors (Table 1). Further, we detected a strong trend toward association of *GATA3-AS1* level with progression-free survival (PFS); however, the difference in PFS between the high- and low-expression groups was not significant (Figure 1E). Nevertheless, 5-year recurrence rate was significantly higher for patients with NMIBC in the high-expression group (P < 0.05; Figure 1F). These results suggest that *GATA3-AS1* expression is increased in BLCA and associated with poor patient prognosis.

### *GATA3-AS1* promotes BLCA cell proliferation

**Figure 2:**
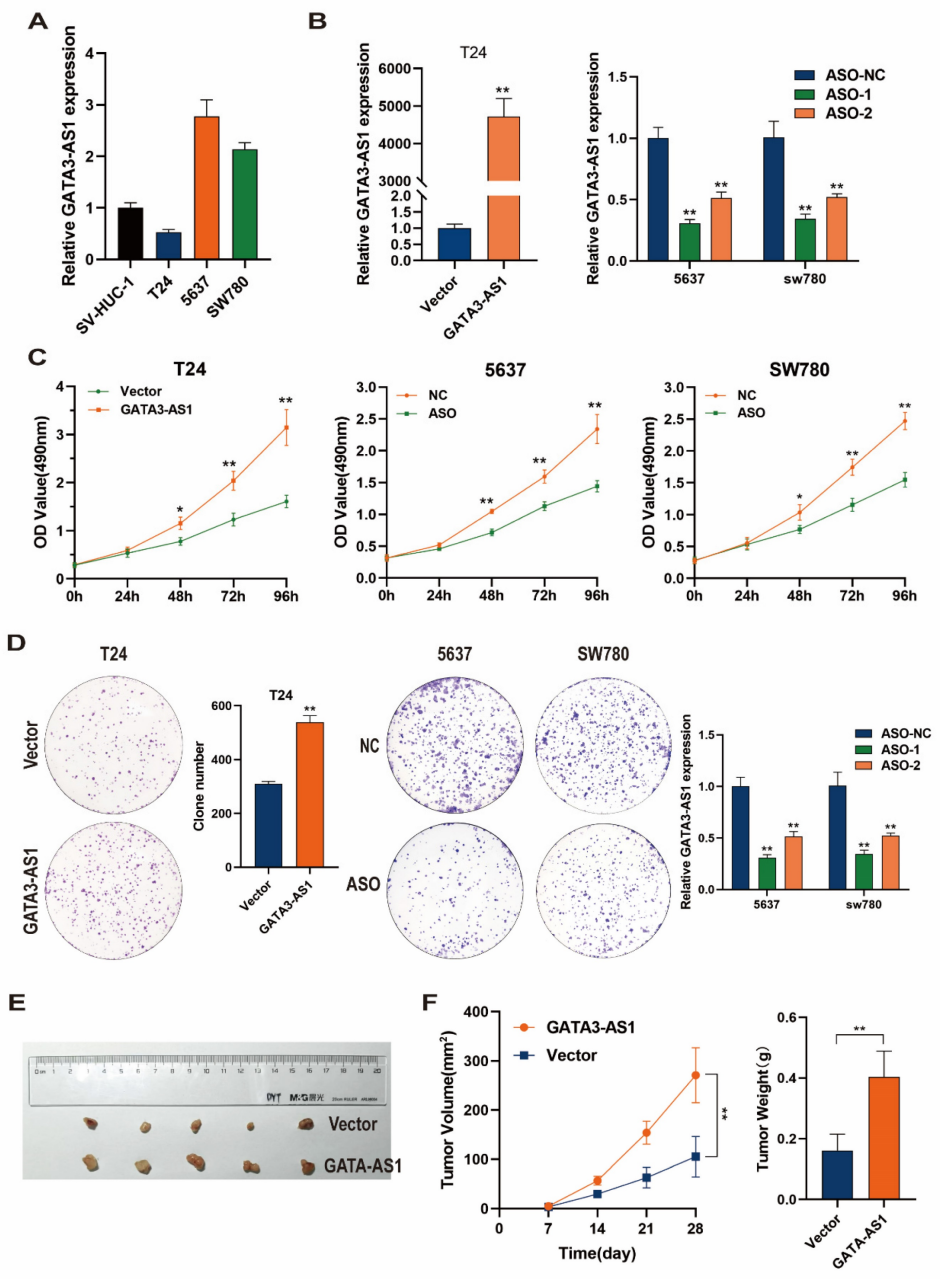
***GATA3-AS1* promoted cell proliferation of BLCA.** (**A**) The expression levels of *GATA3-AS1* in normal bladder epithelial cell (SV-HUC-1) and three BLCA cell lines. (**B**) The efficiency of *GATA3-AS1* knockdown or overexpression was detected by RT-qPCR in the indicated cells. (**C,D**) MTS (**C**) and clone formation assays (**D**) were used to demonstrate the cell proliferation ability of BLCA cells transfected with ASO-*GATA3-AS1* or plasmids. (**E**) Image of subcutaneous xenograft tumor in nude mice with stable overexpression of *GATA3-AS1* in T24 cells. (**F**) Tumor growth curves and weights of different groups. Data are shown as mean ± SD from three independent experiments. ^*^P < 0.05, ^**^P < 0.01.

Levels of *GATA3-AS1* were detected by RT-qPCR in BLCA cells and normal bladder epithelial cells. In contrast to its levels in SV-HUC-1 immortalized human bladder epithelial cells, *GATA3-AS1* was highly expressed in 5637 and SW780 bladder cancer cells, while its levels were low in the T24 bladder cancer cell line (Figure 2A). Next, a *GATA3-AS1* overexpression plasmid of was transfected into T24 cells, while two ASO-*GATA3-AS1* were transfected into 5637 and SW780 cells; overexpression or knockdown efficiencies were detected using RT-qPCR (Figure 2B); knockdown using ASO-1 was effective and was therefore used for subsequent experiments. MTS and colony formation assays revealed that *GATA3-AS1* overexpression significantly increased the proliferation of T24 cells *in vitro*. In contrast, decreasing *GATA3-AS1* levels in 5637 and SW780 cells inhibited their *in vitro* proliferative capacity (Figure 2C, D). To verify these findings *in vivo*, we subcutaneously injected T24 cells stably overexpressing *GATA3-AS1* and control cells into mice. Tumors were dissected out after 4 weeks and their volumes and weights measured. The results suggested that the volume and weight of xenograft tumors were significantly increased in mice injected with cells overexpressing *GATA3-AS1* (Figure 2E, F).

### Alteration of *GATA3-AS1* levels affects BLCA cell invasion and migration

**Figure 3:**
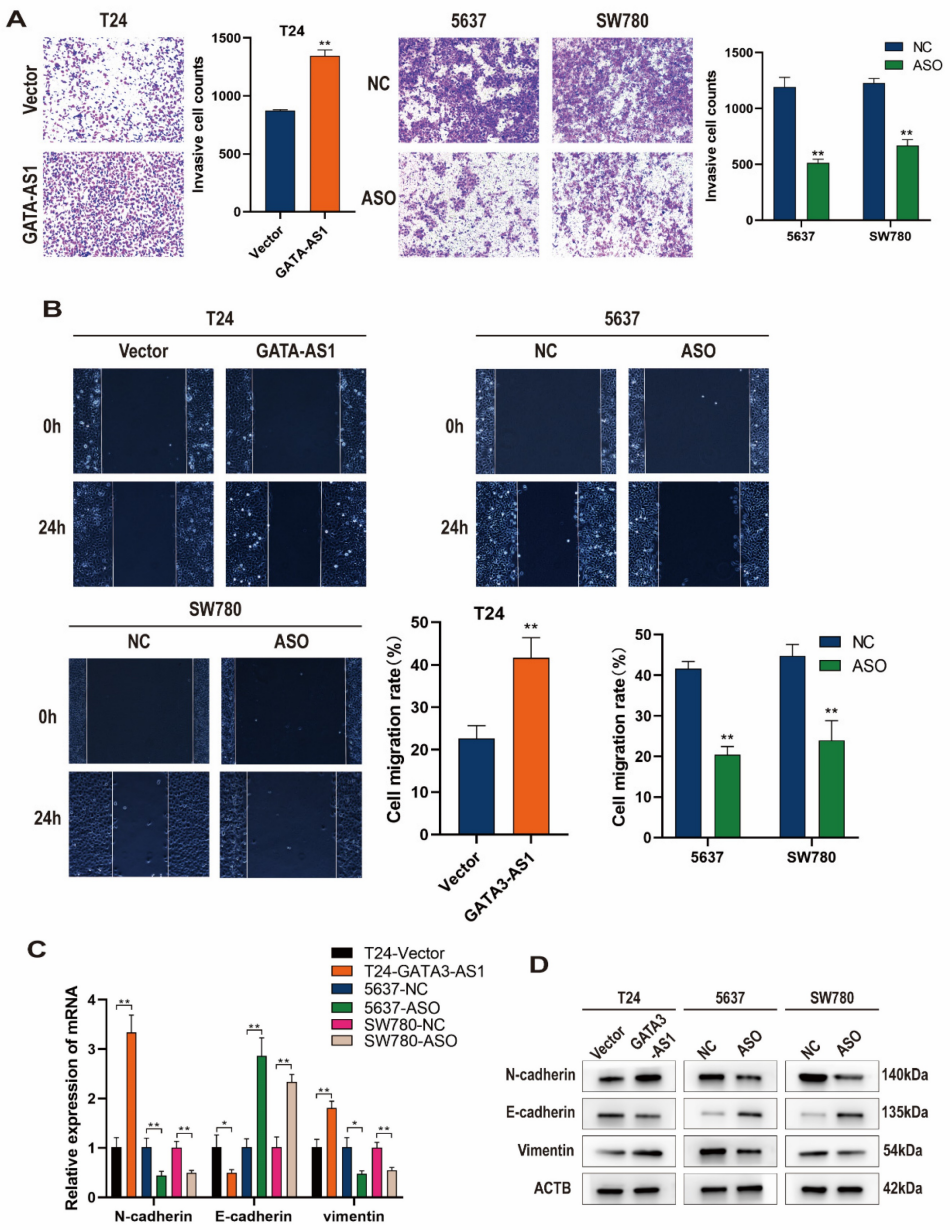
***GATA3-AS1* promotes the migration, invasion, and epithelial-mesenchymal transition (EMT) of bladder urothelial carcinoma (BLCA) cells.** (**A**) The invasion abilities of the indicated cells were assessed by transwell assay. (**B**) Representative images and quantitative data of wound healing migration assay were presented. (**C,D**) Detection of mRNA and protein levels of E-cadherin, N-cadherin, and Vimentin after overexpression and knockdown of *GATA3-AS1* using RT- qPCR (**C**) and western blot (**D**). Data are shown as mean ± SD from three independent experiments. ^*^P < 0.05, ^**^P < 0.01.

We next tested the regulatory effects of *GATA3-AS1* on BLCA cell invasion and metastatic functions using Transwell and wound healing assays. The results showed that T24 cells overexpressing *GATA3-AS1* had higher *in vitro* invasion and migration abilities than control cells, while down-regulation of *GATA3-AS1* in 5637 and SW780 cells reduced their invasion and migration (Figure 3A, B).

Further, changes of cellular EMT markers were detected by RT-qPCR and western blotting. When *GATA3-AS1* was upregulated in T24 cells, N-cadherin and Vimentin mRNA and protein levels were elevated, while E-cadherin expression was reduced. Conversely, silencing of *GATA3-AS1* inhibited N-cadherin and Vimentin mRNA and protein expression, while increasing that of E-cadherin in 5637 cells and SW780 cells (Figure 3C, D). These findings indicate that *GATA3-AS1* dysregulation enhances BLCA cell proliferation and migration and is associated with promotion of the EMT process in BLCA cells.

### *GATA3-AS1* binding to DDX5 helps maintain its stability

**Figure 4:**
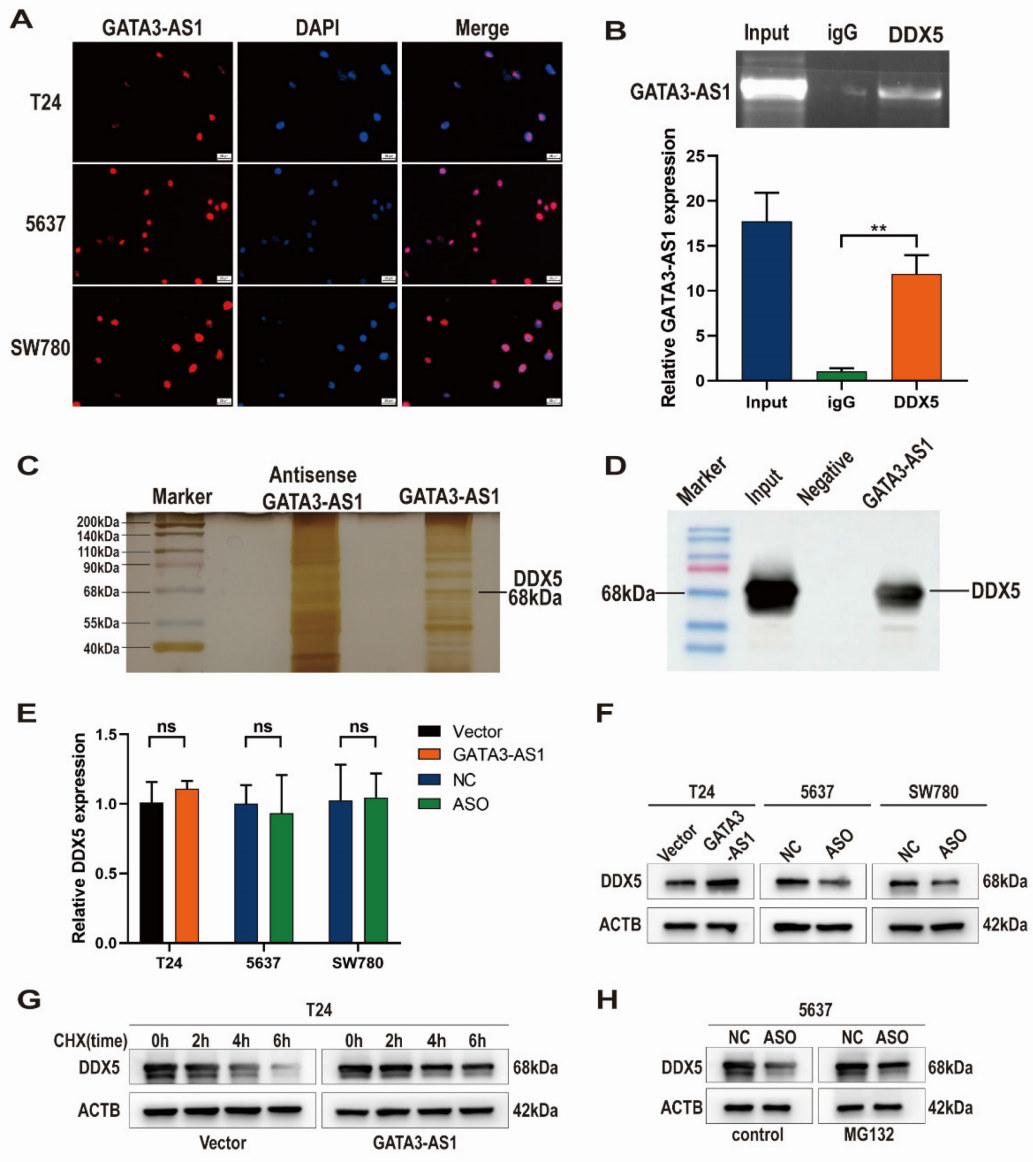
***GATA3-AS1* interacts with DDX5 and enhances its stability.** (**A**) *GATA3-AS1* was identified by FISH to be located within the nucleus of indicated cells. (**B**) RIP assay showed the interaction between *GATA3-AS1* and DDX5 in 5637 cells. (**C**) RNA pull-down assay was performed in 5637 cells, and *GATA3-AS1* binding protein was analyzed by SDS-PAGE gel electrophoresis and silver staining. (**D**) Confirmed by western blot assay that the differential band at 68kDa is DDX5. (**E,F**) The regulatory effect of *GATA3-AS1* on DDX5 expression in indicated cells was detected by RT-qPCR (**E**) and western blot (**F**). (**G**) Western blot was used to measure the levels of DDX5 protein in *GATA3-AS1* overexpressing T24 cells and control cells treated with protein synthesis inhibitor cycloheximide (CHX). (**H**) Western blot was used to determine the effect of treatment with proteasome pathway inhibitor (MG132) on the level of DDX5 in 5637 cells with *GATA3-AS1* knockdown. ^**^P < 0.01. ns.: not significant.

To determine how *GATA3-AS1* promotes BLCA progression, first we analyzed its subcellular localization in BLCA cells by FISH, which showed that *GATA3-AS1* was predominantly concentrated in the nucleus (Figure 4A). We then used an RNA probe for* GATA3-AS1* to screen for proteins interacting with *GATA3-AS1* by RNA pull-down assay, silver staining, and MS, while an RNA probe for the antisense sequence of GATA3-AS1 was used as a negative control, and finally identified DDX5 as a target interacting with *GATA3-AS1*. Western blotting experiments also verified that a specific band migrating at 68 kDa was indeed DDX5 (Figure 4C, D). We next predicted DDX5 subcellular location using the Uniprot website (https://www.uniProt.org), and found that it was also localized to the nucleus, supporting the possibility that *GATA3-AS1* and DDX5 interact. RIP assays further confirmed an interaction between *GATA3-AS1* and DDX5 (Figure 4B).

Subsequently, we further investigated the regulatory effect of the interaction between *GATA3-AS1* and DDX5. No significant change in* DDX5* mRNA was detected after *GATA3-AS1* overexpression or knockdown (Figure 4E). In contrast, levels of DDX5 protein changed in the same direction as those of *GATA3-AS1* level (Figure 4F), suggesting that *GATA3-AS1* may affect DDX5 at the post-translational stage. Therefore, we hypothesized that *GATA3-AS1* can increase DDX5 protein by inhibiting its degradation. To test this notion, we treated T24 cells using a blocker of protein synthesis (cycloheximide), and found that *GATA3-AS1* overexpression slowed DDX5 protein degradation (Figure 4G), whereas *GATA3-AS1* silencing in 5637 cells resulted in a reduction of DDX5, which was alleviated by treatment with the proteasome inhibitor, MG132 (Figure 4H). These results suggest that *GATA3-AS1* binds to DDX5 protein in BLCA cells and increases its levels by enhancing its stability and reducing its degradation.

### *GATA3-AS1* activates the Wnt/β-catenin signaling pathway by targeting DDX5

**Figure 5:**
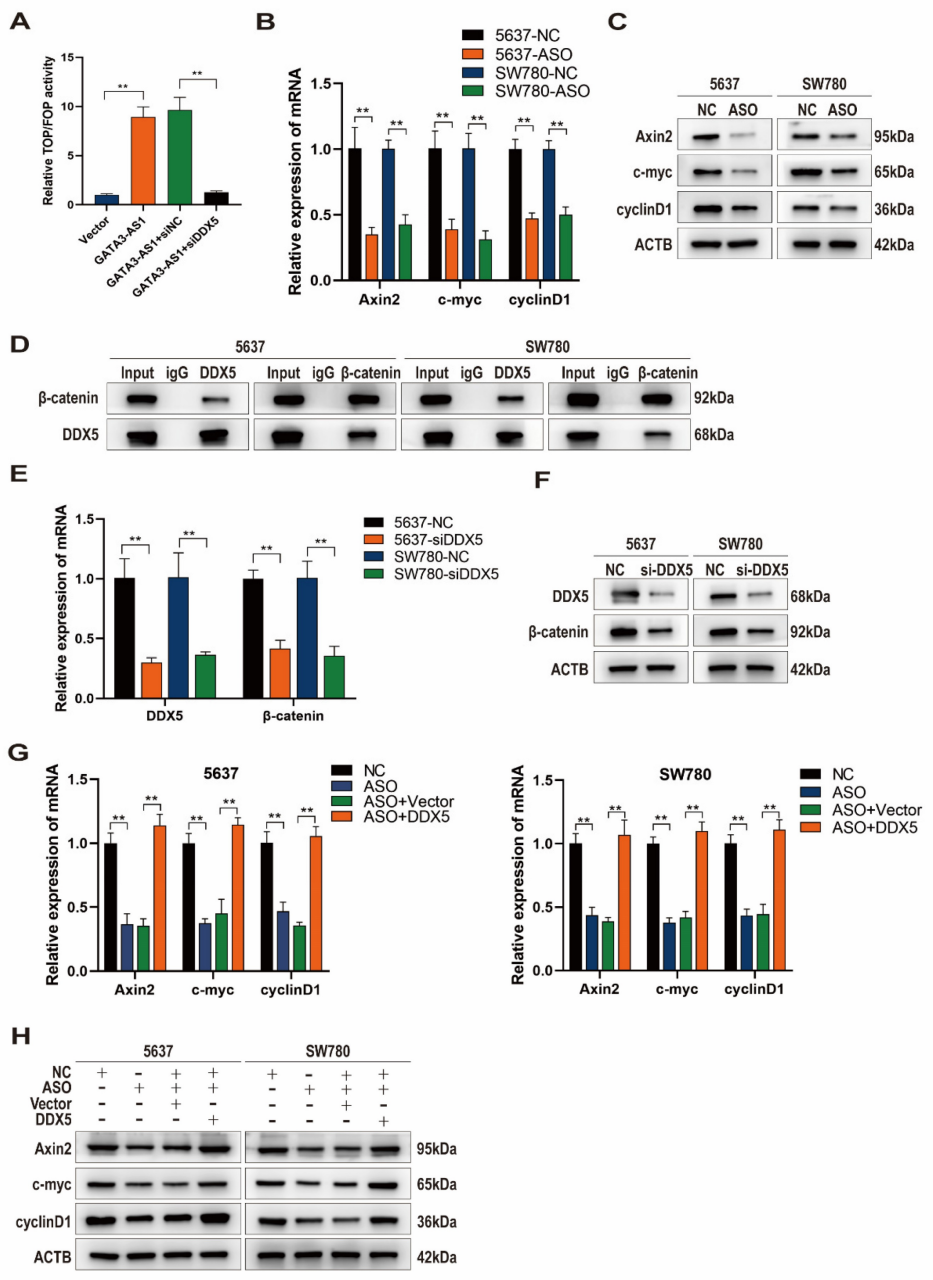
***GATA3-AS1* regulates the Wnt/β-catenin signaling pathway through DDX5 mediation** (**A**) TOP-FOP flash reporter gene assay showed that knocking down *GATA3-AS1* can reduce Wnt/β-catenin transcriptional activity and reverse this result with overexpression of DDX5. (**B**,**C**) Detection of the regulatory effect of *GATA3-AS1* on the expression of key molecules (Axin2, myc, and cyclinD1) in the Wnt pathway in designated cells by RT qPCR (**B**) and western blot (**C**). (**D**) Co- IP determination of the interaction between DDX5 and β-catenin in 5637 cells and SW780 cells. (**E,F**) The regulation effect of DDX5 on β-catenin expression was detected by RT-qPCR (**E**) and western blot (**F**). (**G**,**H**) RT- qPCR (**G**) and western blot (**H**) analysis showed that overexpression of DDX5 reversed the effect of *GATA3-AS1* silencing on the essential proteins of the Wnt/β-catenin signaling pathway (Axin2, myc, and CyclinD1) in designated cells. ^**^P < 0.01.

Previous studies have shown that DDX5 binds to β-catenin protein to form a transcriptional activation complex, which performs important functions in promoting β-catenin transcription, activating the Wnt signaling pathway, and driving the EMT process^22^. Given our previous results suggesting that *GATA3-AS1* affects EMT in BLCA cells, we suspected that *GATA3-AS1* may affect the Wnt/β-catenin signaling pathway via DDX5. *GATA3-AS1* overexpression increased the level of β-catenin-mediated *TCF*/*LEF* transcriptional activity in the Wnt signaling pathway, as determined using the TOP/FOP luciferase reporter gene assay, whereas concomitant DDX5 knockdown abolished this impact (Figure 5A). Axin2, c-Myc, and Cyclin D1 mRNA and protein levels, as detected by RT-qPCR and western blot, were reduced after *GATA3-AS1* silencing, supporting that *GATA3-AS1* has a regulatory effect on the Wnt pathway (Figure 5B, C). β-catenin is a key molecule in activation of the classical Wnt pathway, and Co-IP assay demonstrated that DDX5 and β-catenin could directly bind to one another in 5637 cells and SW780 cells (Figure 5D). Further, DDX5 influenced β-catenin mRNA and protein levels (Figure 5E, F). Moreover, reduction of Wnt pathway signaling molecules by *GATA3-AS1* knockdown was successfully reversed by DDX5 overexpression (Figure 5G,H). In conclusion, *GATA3-AS1* regulates Wnt/β-catenin signaling and DDX5 has an indispensable role in this function.

### DDX5 mediates the effect of *GATA3-AS1* in promoting BLCA progression

**Figure 6:**
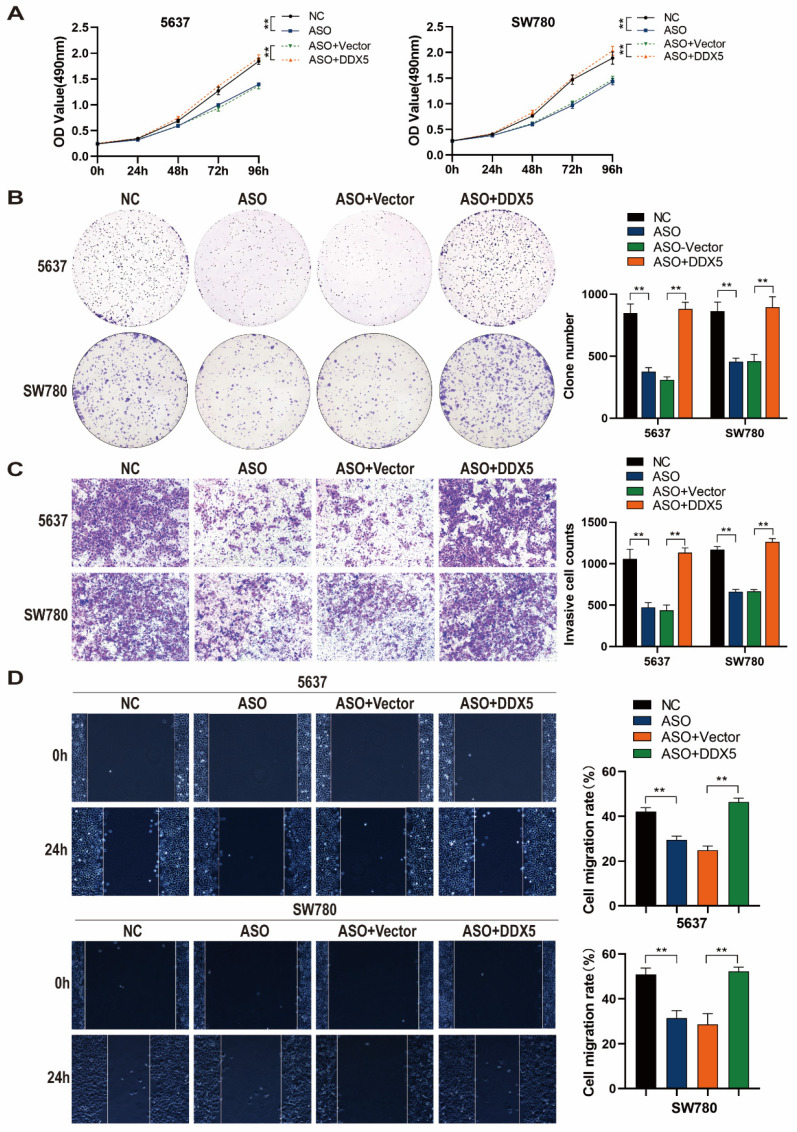
** DDX5 mediates the *GATA3-AS1*-regulated promotion of BLCA progression.** (**A**,**B**) The results of MTS (**A**) and clone formation experiments (B) showed that overexpression of DDX5 reversed the reduced cell proliferation ability caused by downregulation of *GATA3-AS1*. (**C**,**D**) Transwell invasion assay (**C**) and wound healing assay (**D**) showed that overexpression of DDX5 rescued the inhibition of *GATA3-AS1* silencing on BLCA cell invasion and migration ability.

To determine whether *GATA3-AS1* can promote BLCA progression and test if DDX5 is involved in this process, we performed rescue experiments. DDX5 overexpression reversed the inhibitory effect of *GATA3-AS1* knockdown on 5637 and SW780 cell proliferation (Figure 6A, B). In addition, DDX5 overexpression attenuated the reduction of 5637 and SW780 cell invasion induced by *GATA3-AS1* knockdown (Figure 6C). In cells with both *GATA3-AS1* knockdown and DDX5 expression no inhibition of 5637 or SW780 cell migration was detected (Figure 6D). These results indicate that *GATA3-AS1* can promote BLCA progression mediated by DDX5.

## Discussion

Through extensive utilization of bioinformatics technology and high-throughput sequencing, lncRNAs have been identified as vital participants in BLCA progression. The processes of cancer cell growth, migration, apoptosis, glycolysis, and EMT are all regulated by lncRNAs^12^. In addition, lncRNA acquisition and loss influence the response of BLCA cells to chemotherapy with cisplatin, doxorubicin, and gemcitabine, as well as their response to PD1/L1 immunotherapy, demonstrating great potential as molecular markers for predicting prognosis and clinical efficacy^23^-^25^.

In this study, we identified *GATA3-AS1* by searching for lncRNAs abnormally overexpressed in BLCA in online databases. To clarify the value of *GATA3-AS1* analysis in BLCA, we examined its expression BLCA tissues, and found that *GATA3-AS1* overexpression was closely correlated with BLCA depth of invasion and pathological grade. In addition, patients with high *GATA3-AS1* expression had a significantly increased risk of disease recurrence, and this manifestation was more prominent in patients with NMIBC. Although we did not evaluate patient overall survival, our results are sufficient to suggest that *GATA3-AS1* is highly likely to function as a tumor promoter, as supported by the results of cell function assays and detection of EMT-associated molecules.

The relationship between GATA3 and bladder cancer molecular subtype and prognosis has been confirmed; it is an important molecular feature of luminal subtype bladder cancer, and its mutation is related to breast and bladder cancer occurrence^26^-^28^. In bladder cancer, loss of GATA3 can induce EMT and the expression of pro-metastatic molecules, such as MMP-2 and MMP-9, leading to disease progression^29^. As the antisense lncRNA of GATA3, Zhang *et al.* found that *GATA3-AS1* can promote triple-negative breast cancer progression by promoting GATA3 ubiquitination, thereby disrupting GATA3 protein stability^19^. It is reasonable to suspect that the promotion of BLCA EMT by *GATA3-AS1* may be related to GATA3 degradation, however, due to the diversity of molecular mechanisms, the existence of other mechanisms of action in BLCA requires in-depth investigation.

In previous studies, most lncRNAs have been thought to act as molecular sponges in the cytoplasm, which affect the expression of downstream proteins by competitively binding miRNAs^30^; however, the discovery of large numbers of RBPs has gradually revealed interactions between lncRNAs and RBPs. LncRNAs can interact with RBPs at transcriptional or post-transcriptional stages, to regulate their localization, modification, stability, and activity^31^. Based on these theories, we attempted to identify *GATA3-AS1* RBPs by RNA pull-down assay, and discovered DDX5. DDX5 (p68) is a member of the DEAD-box family of RNA helicases, which have varying functions in different cancers^32^. The relationship between DDX5 and the Wnt pathway is particularly close, and β-catenin stability and entry into the nucleus are key to Wnt/β-catenin signaling pathway activation, where DDX5 acts on the promoter region of the gene encoding β-catenin to activate its transcription^22^. Further, direct binding of DDX5 to β-catenin in the cytoplasm enhances β-catenin stability and promotes its nuclear translocation, thereby co-activating the transcription of several oncogenes in the Wnt pathway (encoding c-Myc, CyclinD1, c-Jun, and Fra-1)^33^. In addition, β-catenin directly induces DDX5 expression, forming a positive feedback loop that promotes tumor progression^34^. Wnt/β-catenin is the most important regulator of EMT, and lncRNAs often have major roles influencing the Wnt/EMT axis in human cancers. Based on the above evidence, we proposed and tested a hypothetical *GATA3-AS1*/DDX5/β-catenin axis. Unsurprisingly, *GATA3-AS1* can indeed regulate DDX5 at the protein level, by enhancing its stability, rather than inducing *DDX5* mRNA transcription. Further, *GATA3-AS1* affected β-catenin transcriptional activity via DDX5, which in turn activated the Wnt pathway to regulate BLCA growth and metastasis.

Bladder cancer is a common urinary system tumor with high heterogeneity, showing differentiated genetic characteristics at various stages of tumor development^35^, ^36^. Therefore, although new treatment methods continue to emerge, their effectiveness varies among individuals. In the past decade, several consensus classification systems based on gene expression subtypes have been reported, in an attempt to establish predictive model for patient prognosis and clinical efficacy; however, none of these methods can be applied to clinical decision-making^37^. How to improve the non-invasive monitoring of bladder cancer recurrence through molecular markers, how to predict the response of NMIBC to intravesical adjuvant immunotherapy with Bacillus Calmette-Guerin, and how to predict and improve the response to treatment of locally advanced and metastatic bladder cancer remain the top priorities for the development of precise treatment for bladder cancer^7^. Due to the limitations of available specimens, more studies to explore the guiding significance of the *GATA3-AS1*/DDX5/β-catenin axis in clinical decision-making are needed.

In summary, our study confirms the overexpression and oncogenic role of *GATA3-AS1* in BLCA. Our data reveal that *GATA3-AS1* activates the Wnt/EMT axis, mediated by DDX5, which may be a potential molecular mechanism leading to increased BLCA invasiveness. Our findings provide data on a new molecular indicator with potential to improve the prediction of prognosis for patients with bladder cancer.

## Supplementary Material

Supplementary tables.

## Figures and Tables

**Table 1:** Association between GATA3-AS1 expression and clinicopathological factors in BLCA patients (n=90)

			GATA3-AS1 level		
Parameters	Group	Total	Low (n=45)	High (n=45)	*P* Value
**Age**	≤65	36	20	16	0.389
	>65	54	25	29	
**Gender**	Male	69	35	34	0.803
	Female	21	10	11	
**T stage**	≤T1	59	34	25	0.046*
	T2∼4	31	11	20	
**N stage**	Negative	83	42	41	0.694
	Positive	7	3	4	
**Grade**	Low-grade	63	37	26	0.011*
	High-grade	27	8	19	
