# Supplementary material for: LncRNA GATA3-AS1 Promotes Bladder Uroepithelial Cancer Progression by Stabilizing DDX5
Source: J Cancer. 2025 Jan 1;16(3):835–47. doi: 10.7150/jca.104034 (PMC11705050; doi:10.7150/jca.104034)
Supplement: Supplementary file 1 — Supplementary tables. [file jcav16p0835s1.pdf]

Table 1 Primers for real-time PCR.

Primer name	Forward (5'-3')	Reverse (5'-3')
GATA3-AS1	TTGTTCCCTCTTCGCTCCT	TTGTTCCTTCACCGCATG
E-cadherin	CGAGAGCTACACGTTCACGG	GGCCTTTTGACTGTAATCACACC
N-cadherin	CAACTTGCCAGAAAACTCCAGG	ATGAAACCGGGCTATCTGCTC
Vimentin	CGCCTGCAGGATGAGATTCAG	TCAGGGAGGAAAAGTTTGGAAGA
DDX5	TGAGCGACCTTATCTCTGTGC	GGTCATCCTTCATGCCTCCT
β-catenin	ATGGAACCAGACAGAAAAGCGGC	GCTACTTGTTCTTGAGTGAAG
c-myc	GGACTATCCTGCTGCCAAGA	CGCCTCTTGACATTCTCCTC
cyclin D1	GTGCTGCGAAGTGGAAACC	ATCCAGGTGGCGACGATCT
Axin2	CTGGGGGCAGCGAGTATTAC	GCCTTTCCCATTGCGTTTGG
GAPDH	AGGTGAAGGTCGGAGTCAACG	AGGGGTCATTGATGGCAACA

Table 2 Sequences for ASO or siRNA.

Names	Forward (5'-3')	Reverse (5'-3')
ASO-GATA3-AS1-1	CUUAAAUCUUAAUCCCGGGC	____
ASO-GATA3-AS1-2	UCUCCGCGCGUCAAUCGA	____
ASO-NC	UUCUCCGAACGUCACGU	____
si-DDX5	GCAAGUAGCUGCUGAAUUU	pAUAUUCAGCAGCUACUUGCUU
si-NC	UUCUCCGAACGUGUCACGUTT	ACGUGACACGUUCGGAGAATT
